# The Implication of PGC-1α on Fatty Acid Transport across Plasma and Mitochondrial Membranes in the Insulin Sensitive Tissues

**DOI:** 10.3389/fphys.2017.00923

**Published:** 2017-11-15

**Authors:** Elżbieta Supruniuk, Agnieszka Mikłosz, Adrian Chabowski

**Affiliations:** Department of Physiology, Medical University of Bialystok, Bialystok, Poland

**Keywords:** PGC-1α, FATPs, FABPpm, FAT/CD36, lipid metabolism, insulin sensitive tissues

## Abstract

PGC-1α coactivator plays a decisive role in the maintenance of lipid balance *via* engagement in numerous metabolic processes (i.e., Krebs cycle, β-oxidation, oxidative phosphorylation and electron transport chain). It constitutes a link between fatty acids import and their complete oxidation or conversion into bioactive fractions through the coordination of both the expression and subcellular relocation of the proteins involved in fatty acid transmembrane movement. Studies on cell lines and/or animal models highlighted the existence of an upregulation of the total and mitochondrial FAT/CD36, FABPpm and FATPs content in skeletal muscle in response to PGC-1α stimulation. On the other hand, the association between PGC-1α level or activity and the fatty acids transport in the heart and adipocytes is still elusive. So far, the effects of PGC-1α on the total and sarcolemmal expression of FAT/CD36, FATP1, and FABPpm in cardiomyocytes have been shown to vary in relation to the type of PPAR that was coactivated. In brown adipose tissue (BAT) PGC-1α knockdown was linked with a decreased level of lipid metabolizing enzymes and fatty acid transporters (FAT/CD36, FABP3), whereas the results obtained for white adipose tissue (WAT) remain contradictory. Furthermore, dysregulation in lipid turnover is often associated with insulin intolerance, which suggests the coactivator's potential role as a therapeutic target.

## Introduction

A modern lifestyle associated with physical inactivity and over-nutrition inevitably leads to overweight or obesity and has a critical importance in the development of insulin resistance (IR). Such a deleterious alterations in lifestyle result in tremendous expanse of type 2 diabetes (T2DM). According to the International Diabetes Federation (IDF) in 2015 there were 415 million people suffering from diabetes and IDF predicts that by 2,040 this number will grow to 642 million (Nam et al., 2015). For these reasons, researchers focus on the possible mechanisms contributing to IR and its associated comorbidities as well as on the elaboration of effective treatments.

**Figure 1:**
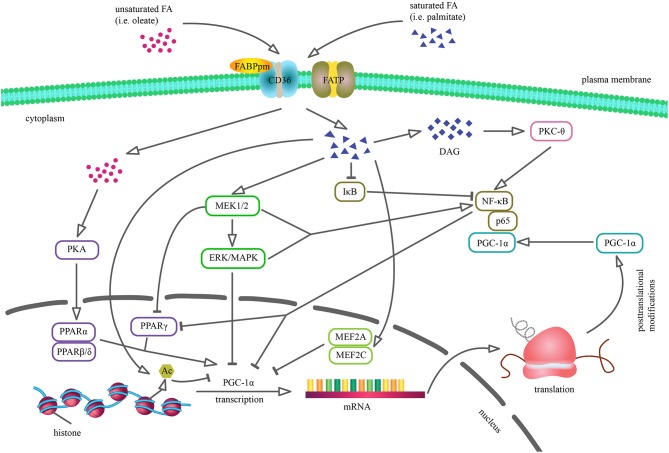
Cellular mechanisms by which saturated and unsaturated fatty acids may alter PGC-1α expression and activity. Palmitic acid incorporation reduces PGC-1α transcription through the activation of ERK/MAPK cascade, reduction of MEF2 transcription factor DNA binding and stimulation of IκB proteasomal degradation. Moreover, saturated FA contribute to MEK1/2-dependent NF-κB stimulation and DAG accumulation that results in PKCθ-mediated NF-κB induction. PGC-1α downregulation *via* NF-κB may involve several processes, including direct repression as p65 subunit of NF-κB is constitutively physically bound to PGC-1α as well as reduced PPARγ activity, because PGC-1α promoter sequence contains PPRE. Palmitate-induced histone deacetylation results in reduced PGC-1α promoter activity and inhibits PGC-1α expression. Conversely, oleate enhances PGC-1α mRNA level *via* PKA-PPARα pathway, thus reversing the coactivator's previous level (Coll et al., 2006, 2008; Crunkhorn et al., 2007; Alvarez-Guardia et al., 2010).

So far, most of the studies suggest that a prolonged increase in plasma non-esterified fatty acid (NEFA) content and subsequent ectopic lipid accumulation interferes with insulin signal transduction and is responsible for the development of IR. Long chain fatty acids (LCFA) plasma concentration has been linked with an enhanced intracellular fatty acid (FA) transport, which exceeds the rate of β-oxidation in a tissue (Serra et al., 2013). As a result, lipid peroxidation and generation of reactive oxygen species (ROS) are induced (Ayala et al., 2014). Nowadays, a lot of data connect lipid-dependent oxidative stress with IR etiology (Schrauwen et al., 2006), for instance 4-hydroxynonenal (4-HNE) reduces protein kinase B (PKB/Akt) and Akt substrate of 160 kDa (AS160) phosphorylation as well as insulin receptor substrates (IRS-1 and IRS-2) expression (Prasannarong et al., 2014). Furthermore, the impaired lipid utilization, characterized by increased triacylglycerols (TAG) and other bioactive lipid fractions levels, such as fatty acyl CoA, diacylglycerols (DAG) and ceramides (CER), admittedly contributes to adverse alternations in insulin signaling pathway (Erion et al., 2016). Briefly, DAG stimulate protein kinase C (PKC-θ, PKC-βII, and PKC-δ isoforms) that in turn dephosphorylates IRS-1, thereby blocking phosphatidylinositol-3 kinase (PI3K) activation (Szendroedi et al., 2014; Łukaszuk et al., 2015a). Excessive accumulation of CER induces phosphatidyl phosphatase 2A (PP2A) and protein kinase C λ/ζ (PKCλ/ζ), thus inhibiting the phosphorylation of PKB/Akt in the position of Thr308 and Ser473 (Hage Hassan et al., 2014; Kurek et al., 2015). Additionally, CER and DAG can upregulate nuclear factor κB (NF-κB) level (Figure 1) (Coll et al., 2008; Hommelberg et al., 2011), which is involved in the activation of inflammatory processes, including IL-6 secretion that impairs insulin action at the level of IRS-1 (Nieto-Vazquez et al., 2008). The CER-mediated induction of signaling pathway of the protein kinase inhibitor complex of IκB and protein kinase c-Jun leads to an inhibition of tyrosine phosphorylation of IRS1 (Boon et al., 2013). Generally, high lipid availability, mitochondrial dysfunction, ROS and lipid intermediates accumulation may induce IR. In the light of the above mentioned statements, understanding of the mechanisms responsible for the control of FA uptake and metabolism is essential for the discovery of potential therapeutic options.

## PGC-1α as a pivotal factor in lipid balance

The peroxisome proliferator-activated receptor coactivator-1 (PGC-1) family serves as a major regulator of cellular metabolism by activation of a wide range of nuclear receptor (NR) and non-NR transcription factors. The first discovered member of the PGC-1 family was a 91 kDa nuclear protein (Besseiche et al., 2015) identified in brown adipose tissue (BAT) in cold-induced thermogenesis studies and termed PPARγ coactivator 1α (PGC-1α) (Puigserver et al., 1998). A sequence analysis revealed that PGC-1α (also known as PPARGC1A) is composed of several distinct domains, including an amino-terminal activation domain covering nuclear receptor docking region, a central regulatory sequence and a carboxy-terminal RNA recognition motif (Martínez-Redondo et al., 2015). The biological activity of PGC-1α is tightly controlled by transcriptional (multiple promoter regions, alternative splicing) and post-translational (phosphorylation, acetylation or methylation) modifications, which give rise to PGC-1α-a, PGC-1α-b, PGC-1α-c, and NT-PGC-1α mRNA isoforms involved in the cellular adaptation to the environmental conditions (Popov et al., 2015). The dynamic changes in PGC-1α activity also depend on its interactions with other coregulators/coactivators functioning as sensors of cellular energy status. A set of genes involved in energy balance remains under the antagonistic control of PGC-1α and receptor-interacting protein 140 (RIP140) (Kupr and Handschin, 2015). PGC-1α sensitivity to RIP140-mediated repression increases after SUMOylation by small ubiquitin-like modifier (SUMO-1) protein, which reduces transcriptional activity of the coactivator without an impact on its stability and cellular localization (Rytinki and Palvimo, 2009). Additionally, retinoblastoma protein (pRB) promoting white adipose tissue (WAT) development represses PGC-1α promoter activity and diminishes the coactivator's transcription rate (Scimè et al., 2005; Váraljai et al., 2015). In adipocytes PGC-1α is also controlled by transcriptional coregulator PRDM16 involved in attenuation of white fat-selective genes expression. PGC-1α competes with C-terminal binding protein (CtBP) for binding to PRDM16 and in such complex strongly stimulates the expression of brown fat genes, including PGC-1α itself (Kajimura et al., 2008). Moreover, CREB-regulated transcription coactivator (CRTC2) was found to positively stimulate PGC-1α transcription in skeletal muscle cells, whereas dysregulation of CRTC2/CREB pathway in diabetic rats may contribute to a reduced PGC-1α level (Rahnert et al., 2016). The half-life time of the protein is about 20 min and afterwards it undergoes ubiquitylation and proteasomal degradation (Sano et al., 2007). PPARβ can limit PGC-1α degradation *via* binding to the coactivaor and reducing its ubiquitination in skeletal muscle challenged by exercise (Koh et al., 2017). Recent studies performed *in vitro* on muscle and liver cells discovered a new regulator of PGC-1α activity, namely flavoprotein NADH quinone oxidoreductase 1 (NQO1). NQO1 binds to PGC-1α and protects the coactivator from ubiquitin-independent proteasomal degradation executed by the 20S proteasome catalytic particle in NADH-dependent manner (Adamovich et al., 2013). What is also important, PGC-1α does not directly bind to DNA, but interacts with a variety of transcription factors and therefore mediates chromatin remodeling (*via* CBP/p300 and SRC-1) and DNA transcriptional machinery activation (*via* TRAP/Mediator), eventually regulating gene expression (Wallberg et al., 2003; Sano et al., 2007). Finally, it coordinates such processes as mitochondrial biogenesis and oxidative metabolism (Popov et al., 2015). Therefore, PGC-1α is the most important powerful lipid homeostasis regulator, however efforts for understanding its engagement in LCFA metabolism are still in their infancy.

PGC-1α is abundantly expressed in tissues with high energy requirements, such as heart, skeletal muscle and BAT (Liang and Ward, 2006; Vega et al., 2015). Moreover, its expression in subcutaneous and visceral adipose tissue is significantly lower than in skeletal muscle (Heilbronn et al., 2012). Multiple factors enhance muscular PGC-1α content, most notably an increased contractile activity (Irrcher et al., 2003; Mathai et al., 2008), which also stimulates translocation of PGC-1α specifically to subsarcolemmal (SS) mitochondrial subpopulation in a process mediated by AMP-activated protein kinase (AMPK) (Smith et al., 2013). Furthermore, exercise and β_3_-adrenergic signaling were shown to strongly induce PGC-1α expression in BAT (Ruschke et al., 2010; Kim et al., 2016).

Plasma free fatty acids (FFA) level is responsible for the modulation of tissue PGC-1α expression as evidenced by inverse correlation between PGC-1α mRNA content in human skeletal muscle and FFA concentration (Richardson et al., 2005). Consistent with this notion, the reduced PGC-1α transcription has been observed in skeletal muscle (Crunkhorn et al., 2007) and adipose cells (Semple et al., 2004) of obese subjects. However, transcriptional control is often not readily evident as there are numerous observations showing a poor relationship between PGC-1α mRNA and protein expression (Watt et al., 2004; Sparks et al., 2005). The *in vitro* studies on myocytes revealed the opposite impact of saturated and unsaturated FA on PGC-1α expression and activity. Palmitate reduced PGC-1α level in a process mediated by mitogen-activated protein kinase (MAPK) and NF-κB, whereas oleate administration prevented the palmitate-induced PGC-1α downregulation (Figure 1) (Coll et al., 2006, 2008; Crunkhorn et al., 2007; Alvarez-Guardia et al., 2010). It is worth to emphasize that these observations may not reflect the physiological or pathophysiological *in vivo* effects of FA, since palmitic and oleic acids constitute only a part of FFA conglomerate present in the blood plasma. Contradictory results were observed in Huh7 hepatocytes as exposure to palmitic acid resulted in a dose and time dependent increase in PGC-1α expression. Additionally, the upregulation in hepatic PGC-1α level was demonstrated in mice with non-alcoholic fatty liver disease, suggesting different role of the coactivator in response to FA treatment in skeletal muscle and the liver (Maruyama et al., 2016). Understanding the tissue specific functions of the coactivator has been made possible thanks to both gain- and loss-of-function models. Therefore, our review focuses on the comparison and assessment of the lipid metabolism features in both the model types (Tables 1, 2).

**Table 1:** The effects of PGC-1α overexpression on the lipids metabolism.

**Experimental model**	**PGC-1α status**	**Lipid metabolism features**	**References**
**SKELETAL MUSCLE**			
MPGC-1α TG mice	Overexpression	↑ mRNA for genes involved in FA transport (FAT/CD36, FABP3, FATP1, CPT1b), FA oxidation (MCAD, LCAD, VLCAD, PDK4), oxidative phosphorylation (Cyt *c*, CoxIV, UQCRB) and TCA cycle (Idh3α) ↑ (+166%) mtDNA ↑ (+57.6%) glycogen stores in the fed state	Calvo et al., 2008
MPGC-1α TG mice (6 weeks on HFD)	Overexpression	↑ proteins involved in FA transport (FAT/CD36, FABP3, CPT1b), β-oxidation (ACADs, ETFA, HADH) and TCA cycle (CS, Idh3b, ETFA) in isolated mitochondria ↑ rate of [^14^C]palmitate complete oxidation to CO_2_ and incomplete oxidation to ASM ↑ rate of absolute β-oxidation modestly ↑ DAG no differences in CER content robustly ↑ acylcarnitines and acyl-CoAs levels	Wong et al., 2015
C2C12 myotubes	PGC-1α overexpression using adenoviral vectors (mRNA ↑ 86-fold, protein ↑ 8.5-fold)	↑ FA oxidation (≈+31%) ↓ palmitate uptake (−6%) no differences in lipid synthesis from glucose in the basal or insulin-stimulated states ↑ mRNA for lipid oxidation: CPT1b (13.1-fold), malonyl-CoA decarboxylase (+50%) and ACADs (+69-213%) ↓ acyl-CoA oxidase 1 (−22%) ↑ mRNA for lipid synthesis: ↑ ACC1 (cytosolic) (≈+30%), ACC2 (≈+500%), FAS (≈+45%), CS (≈+212%) and DGAT1 (≈+170%) no differences in total lipid content *24 h incubation with [^13^C]glucose:* ↑ FA content (+3.1–4.6 fold) ↑ C16, C18, C18:1 and C18:2 CER content, no differences in sphingomyelin content *Palmitate treated cells:* serum-replete conditions: ↑ total lipid content, ↑ TAG, ↑ DAG serum-starved conditions: ↓ total lipid content (≈-42%), mainly *via* ↓ phospholipid fraction (≈-41%), ↓ DAG (−43%) *Oleate treated cells:* serum-replete conditions: ↑ total lipid content, ↑ TAG, ↓ tendency to TAG accumulation serum-starved conditions: no significant differences	Espinoza et al., 2010
Male Sprague-Dawley rats	Modest PGC-1α overexpression (mRNA +28%, protein + 24%)	↑ CS activity (+13% in red and white muscle, mtDNA +13%, activity altered within IMF, but not SS mitochondria) ↑ FAT/CD36 protein expression (red muscle +35%, white muscle +195%) no differences in FABPpm and HSL proteins expression no differences in intramuscular TAG content ↑ COXIV protein expression (red muscle, SS +15%, IMF +33%; white muscle, SS + 75%, IMF +28%) ↑ CPTI protein in red muscle SS mitochondria (+35%) and ↓ in white muscle IMF mitochondria (-20%), CPTI activity not altered ↑ FAT/CD36 protein in SS mitochondria (red muscle +17%; white muscle +15%), but no changes in IMF mitochondrial ↑ mtTFA protein expression in red muscle (SS +15%; IMF +37%) and white muscle (SS +85%; IMF +25%) ↑ palmitate oxidation in SS mitochondria (red muscle +116%, white muscle +40%), no changes in palmitate oxidation in IMF mitochondria	Benton et al., 2008
MPGC-1α TG mice	≈9 times higher PGC-1α expression	↑ mRNA for CPT1b (+237%), MCAD (+169%), CS (+299%) ↑ mRNA for ACC2 (+189%) ↑ mRNA for OXPHOS system genes (subunits of complexes I - V) ↑β-oxidation rate (+62%)	Summermatter et al., 2011
L6 myotubes	PGC-1α overexpression using adenoviral expression system	↑ mRNA for genes involved in FA metabolism (FABP3, FABP4, FATP1, CPTI, ACADs, PDK4, ACAA2), oxidative phosphorylation (Cyt *c*, Cox6a2, ATP5g1) and TCA cycle (CS, IDH3a, MDH1) ↑ rate of complete (CO_2_) in comparison to incomplete (ASM) β-oxidation	Koves et al., 2005b
MPGC-1α TG mice	≈6-fold increase in gene expression of PGC-1α	↑ expression of OXPHOS and mitochondrial genes (Ndufs1, Ndufv2, Cyt *c*, Cox5b, APT5o) ↑ muscle TAG, lysophosphatidic acid and long-chain acyl CoAs content after HFD ↑ membrane/cytosol ratio of DAG (≈+50%) ↑ PKCθ activity ↑*V*_ATP_ (+50-60%) ↑ mRNA for CPT1, CPT2, VLCAD, LCAD, MCAD ↑ gene and protein expression of ACC2 ↑ phospho-ACC2 level ↑ mRNA for FAT/CD36, DGAT1 and mtGPAT	Choi et al., 2008
MPGC-1α TG mice	Overexpression	*HFD + sedentary conditions:* ↑ mRNA for FAT/CD36, LPL, CPT1b, MCAD, ACC2 ↑ mRNA and activity of CS no significant differences in TAG and CER content ↑ DAG, sphingosine, phosphatidylcholine and phosphatidylathanolamine content ↑ acetylcarnitine level *HFD + training conditions:* no additional affects on mRNA for FAT/CD36, LPL, CPT1b, MCAD, ACC2, CS no additional effect on DAG, CER, phosphatidylcholine and phosphatidylathanolamine content ↑ TAG content ↓ sphingosine and acetylcarnitine content	Summermatter et al., 2013
MPGC-1α TG mice	Overexpression	↑ FAS protein expression (+50%) and activity (+131%) ↑*de novo* lipogenesis (+44%) ↑ IMCL content (+157%) ↑*de novo* synthesized FFA and TAG ↑ FAS mRNA and activity ↑ mRNA for FAT/CD36, FABPpm and FATP4 no changes in mRNA for FATP1, FATP3, FATP6, LPL and ACS ↑ mRNA for DGAT1 (+51%) and mtGPAT (+150%)	Summermatter et al., 2010
Lean and obese Zucker rats	↑ PGC-1α: *lean:* mRNA +31%, protein +20% *obese:* mRNA +37%, protein +27%	↓ TAG content in obese animals (−60%), but ↑ in lean animals (+31%) ↓ DAG (−20%) and ceramide content (−28%) in obese Zucker rat ↑ FAT/CD36 protein levels (lean +25%, obese +16%) ↑ palmitate oxidation in SS mitochondria (lean +37% and obese +18%), not altered in IMF mitochondria in lean or obese Zucker rats	Benton et al., 2010
**HEART**			
Three to 5 months old 129/SvJ mice	PGC-1α overexpression using retroviral expression system	↑ mRNA for nuclear genes encoding mitochondrial (M-CPT I, MCAD) and peroxisomal (ACO) FAO enzymes during the fast ↑ protein expression of CS, β and c subunits of F_1_-F_0_ ATP synthase, COX subunits IV, Va, Vb, Cyt *c*, COX subunit I	Lehman et al., 2000
PGC-1α TG mice	PGC-1α overexpression using retroviral expression system	↑ mRNA for NRF-1 and mtTFA ↓ endogenous PGC-1α expression no differences in mRNA for mitochondrial FAO enzymes (MCAD and CPT I)	Duncan et al., 2007
**ADIPOCYTES**			
3T3-L1 preadipocytes	PGC-1α overexpression using retroviral expression system	modest ↑ mRNA for mitochondrial FAO enzymes (MCAD, LCAD and CPT I) ↓ mRNA for PPARγ ↑^14^CO_2_ production following a 6 h incubation with [1-^14^C]palmitate	Vega et al., 2000

*ACAA2, acetyl-CoA acyltransferase 2; ACADs, acyl-CoA dehydrogenases; ACC2, acetyl-CoA carboxylase; ACO, acyl-CoA oxidase; ACS, acetyl-CoA synthase; ATP5g1, ATP synthase subunit c, isoform 1; ATP5o, ATP synthase; ASM, acid-soluble metabolites; Cox5b, cytochrome c oxidase subunit 5B; Cox6a2, cytochrome c oxidase subunit Via; CoxIV, cytochrome c oxidase subunit IV; CPTI, carnitine palmitoyltransferase I; CS, citrate synthase; Cyt c, cytochrome c, somatic; DGAT1, diacylglycerol acyltransferase 1; ETFA, electron transfer flavoprotein, alpha subunit; FABP3, heart-type fatty acid binding protein; FABP4, adipocyte FABP; FAS, fatty acid synthase; HADH, hydroxyacyl-coenzyme A dehydrogenase; HSL, hormone-sensitive lipase; Idh3α, isocitrate dehydrogenase [NAD] subunit alpha, mitochondrial; LCAD, long-chain acyl-CoA dehydrogenase; LPL, lipoprotein lipase; MCAD, medium-chain acyl-CoA dehydrogenase; MDH1, malate dehydrogenase 1; mtDNA, mitochondrial DNA; mtGPAT, mitochondrial glycerol-3-phosphate acyltransferase; mtTFA, mitochondrial transcription factor A; Ndufs1, NADH:ubiquinone oxidoreductase core subunit S1; Ndufv2, NADH:ubiquinone oxidoreductase core subunit V2; NRF-1, nuclear respiratory factor 1; PDK4, pyruvate dehydrogenase kinase isoform 4; UQCRB, ubiquinol-cytochrome c reductase binding protein; VLCAD, very long-chain acyl-CoA dehydrogenase*.

**Table 2:** The effects of PGC-1α downregulation on the lipids metabolism.

**Experimental model**	**PGC-1α status**	**Lipid metabolism features**	**References**
**SKELETAL MUSCLE**			
C2C12 myotubes	siRNA-mediated knockdown (mRNA for PGC-1α ↓ by 90%)	↓ mRNA for CPT1b, ERRα and complex IV Cox5b trends to ↓ mRNA for ACC2 and CS	Espinoza et al., 2010
PGC-1α^−/−^ mice	Knockout	↓ mRNA for mtTFA ↓ mRNA for genes involved in mitochondrial electron transport (Cyt *c*, CoxIV) and oxidative phosphorylation (β subunit of ATP synthase)	Leone et al., 2005
L6 myotubes	Modest (−24%) PGC-1α protein depletion	↓ Cyt *c* (-16%) and β-HAD (-19%) protein expression ↑ TAG content (+74%) no differences in DAG and CER levels ↑ unsaturated FA among TAG fraction no significant differences in FAT/CD36 (+2%), FATP1 (+4%), FATP4 (+7%) protein expression no differences in palmitate uptake	Lukaszuk et al., 2015b
Skeletal muscle restricted PGC-1α knockout mice	↓ (≈-7–13-fold) mRNA for PGC-1α reduction	↓ mRNA for ERRα, GABPA no significant differences in mRNA for NRF-1 and mtTFA ↓ mRNA for OXPHOS genes (Cyt *c*, Cox5b, APT5o) and electron transport chain genes (Ndufb5, Ndufs1) ↓ fat mass and body fat percentage shift from glucose toward fat metabolism no significant differences in NEFA and total TAG content in the blood	Handschin et al., 2007
**HEART**			
Human myocardium and mouse models	Cyclin T1/Cdk9-dependent PGC-1α suppression (mRNA −60%)	↓ mRNA for Cox1 (-47%), cytochrome *b* (-45%), ATP synthase 8 (-43%) ↓ activities of mitochondrial enzymes: succinate dehydrogenase (complex II), succinate cytochrome *c* reductase (complex II+III), NADH dehydrogenase (complex I), NADH cytochrome *c* reductase (complex I+III) and COXIV no differences in CS activity PGC-1α restoration reversed downregulation of mitochondrial genes	Sano et al., 2004
PGC-1α KO mice	Knockout	↓ (-30%-50%) mRNA for Cyt *c*, Cox5b, ATP5o, MCAD, CPT-1, CPT-2, PPAR-α, PPAR-γ, ERR-α, mtTFA ↓ mRNA for FAT/CD36 (≈-30%) ↓ protein expression of Cyt *c* and ND4L ↓ activity of COX and CS (>−30%) ↓ ATP levels (-20%)	Arany et al., 2005
**ADIPOCYTES**			
PGC-1α KO mice	Adipocyte-restricted PGC-1α knockout	no significant changes in mRNA for genes of the OXPHOS system (Ndufb9, Ndufa9, CoxII, CoxIV, Cyt *c*, ATP5B, ATP5A1), FA oxidation (CPT-1a, MCAD) in WAT no significant changes in mRNA for genes of FA transport (FAT/CD36), TAG synthesis (FAS, DGAT, PEPCK) and hydrolysis (LPL, ATGL) in WAT after exercise ↓ adipocyte size, ↓ WAT mass	Pardo et al., 2011
PGC-1α^−/−^ mice	No PGC-1α protein detected in the nuclear extract prepared from PGC-1α*^−/−^* brown fat	abundant accumulation of large lipid droplets ↓ body fat content after 16 week HFD (≈-17%) in comparison with PGC-1α^+/+^ ↓ mRNA for ERRα, FABP3, Cox7a1	Lin et al., 2004
Preadipocytes isolated from PGC-1α KO mice and stimulated to differentiate	No PGC-1α in mature brown adipocytes	↓ mRNA for ATPase F1 alpha1, CoxIII, CoxII, Cox4i, Cox5b, Cyt *c*	Uldry et al., 2006
PGC-1α KO mice	Adipocyte-restricted PGC-1α deletion	*Adipose tissue lacking of PGC-1α expression:* ↓ mRNA for Cyt *c*, Cox5b, Ndufs5b, Idh3α, CS, CPT1b, PDK4 in WAT ↓ mRNA for FABP3 in BAT and WAT (≈-50%) *HFD conditions:* ↓ mRNA (-30-40%) for genes involved in FFA and TAG uptake and breakdown (FAT/CD36, LPL and LipA) no differences in mRNA for FATP1 ↓ mRNA for PDK4 in WAT ↑ circulating FFA and TAG ↓ mRNA for Cox5b, Idh3α no differences in mRNA for CS, Cyt *c*, ATPase F1a trend toward lower mRNA for FAT/CD36 and LPL in IWAT ↓ mRNA for enzymes involved in adipose lipid synthesis (DGAT, LXR and SCD1 in BAT; LXR, SCD1 and SREBP1 in EWAT)	Kleiner et al., 2012

*ACC2, acetyl-CoA carboxylase; ATGL, adipose triglyceride lipase; APT5o, ATP synthase; ATPase F1 alpha1, ATP synthase, F1 complex, alpha subunit; BAT, brown adipose tissue; β-HAD, β-hydroxyacyl-CoA dehydrogenase; Cox5b, cytochrome c oxidase subunit 5B; Cox7a1, cytochrome c oxidase subunit 7a1; CoxIII, cytochrome c oxidase subunit III; CoxIV, cytochrome c oxidase complex IV; CS, citrate synthase; Cyt c, cytochrome c; DGAT, diacylglycerol acyltransferase; ERRα, estrogen-related receptors α; EWAT, epididymal white adipose tissue; FABP3, heart-type fatty acid binding protein; FAS, fatty acid synthase; GABPA, GA-binding protein A; Idh3α, isocitrate dehydrogenase [NAD] subunit alpha, mitochondrial; IWAT, inguinal white adipose tissue; KO, knockout; LipA, lipoprotein(a); LXR, liver X receptors; mtTFA, mitochondrial transcription factor A; ND4L, NADH dehydrogenase, subunit 4L; Ndufb5, NADH dehydrogenase (ubiquinone) 1 beta subcomplex, 5; Ndufs1, NADH:ubiquinone oxidoreductase core subunit S1; NRF-1, nuclear respiratory factor 1; PDK4, pyruvate dehydrogenase kinase isoform 4; PEPCK, phosphoenolpyruvate carboxykinase; SCD1, stearoyl-CoA desaturase-1; SREBP-1, sterol regulatory element-binding protein 1; WAT, white adipose tissue*.

So far, most of the studies indicate that PGC-1α regulates the expression of genes controlling both lipid oxidation and synthesis. Such pleiotropic effects of the coactivator result from its interaction with multiple agents in diverse tissues. Among transcription factor targets of PGC-1α are PPARα, PPARβ/δ and PPARγ, which coordinate the expression of mitochondrial genes as well as indirectly participate in FA transport and utilization (Lin et al., 2005). Furthermore, PGC-1α upregulates the expression of several genes of the tricarboxylic acid (TCA) cycle (Hatazawa et al., 2015) and mitochondrial FA oxidation (FAO) pathway (Calvo et al., 2008). Accordingly, improvement in both FAO and TCA cycle flux in PGC-1α overexpressing cells coincides with a significant increase in the oxidation of palmitate (Espinoza et al., 2010; Wong et al., 2015). Benton et al. demonstrated that this effect was connected with an increase in FAO in SS, but not in intramyofibrillar (IMF) mitochondria, in both insulin sensitive and IR groups (Benton et al., 2008). Importantly, PGC-1α regulates the expression of nuclear and mitochondrial genes that encode the components of electron transport system and oxidative phosphorylation (OXPHOS) *via* NRF-1 and NRF-2 (nuclear respiratory factor 1 and 2), and estrogen-related receptor α (ERRα) coactivation. This results in increased mitochondrial transcription factor A (mtTFA) expression, which is known to control mtDNA replication and transcription and therefore regulates cellular oxidative metabolism (Dillon et al., 2012). Consequently, the augmented expression of cytochrome *c*, cytochrome *c* oxidase (COX) subunits II and IV, and ATP synthase is also a result of PGC-1α action (Table 1) (Lehman et al., 2000; Choi et al., 2008; Espinoza et al., 2010; Smith et al., 2013). Another noteworthy fact is that PGC-1α can stimulate peroxisomal activity and concurrent long-chain and very-long-chain fatty acid oxidation (Huang et al., 2017). Briefly, PGC-1α expression is positively correlated with cellular capacity to completely oxidize FA that in turn reduces intramuscular lipids deposition and improves insulin sensitivity of tissues.

On the other hand, real-time PCR and photometric methods revealed that PGC-1α promotes also lipogenesis in skeletal muscle by increasing the expression and activity of fatty acid synthase (FAS), a multifunctional enzyme engaged in *de novo* lipid synthesis (Espinoza et al., 2010; Summermatter et al., 2013). Chromatin immunoprecipitation assay showed that the mechanism for this effect involves liver X receptor α (LXRα) coactivation that stimulates PGC-1α binding to the liver X receptor-responsive element (LXRE) in FAS promoter. Additionally, muscle specific PGC-1α transgenic (MPGC-1α TG) mice demonstrated exacerbated rates of *de novo* synthesized FFA as well as FA esterification and TAG accumulation (Summermatter et al., 2010). Nevertheless, PGC-1α overexpression does not change the level of acetyl-CoA synthase (ACS), an enzyme implicated in linking the imported lipids to coenzyme A. Concomitantly, despite the enhanced expression of acetyl coenzyme A carboxylase 2 (ACC2), there is no difference in AMPK activity between wild type and transgenic animals (Espinoza et al., 2010). Moreover, the coactivator-dependent intramyocellular lipid (IMCL) accumulation (Summermatter et al., 2010) is associated with impaired glucose homeostasis and regarded as a risk factor for the development of IR and/or T2DM (Mikłosz et al., 2017). However, endurance-trained athletes also display elevated IMCL level combined with high insulin sensitivity, a phenomenon known as “an athletes” metabolic “paradox” (Bergman et al., 2010). In particular, the elevated total DAG level found in trained individuals implies a great role of subcellular localization and composition of lipid fractions in IR development (Amati et al., 2011). Since lipotoxic intermediates are crucial for insulin action deterioration, it is also important to notice that PGC-1α overexpression causes a reduction in intramuscular TAG (−60%), DAG (−20%), and CER (−28%) concentration in obese Zucker rats. On the other hand, an increase in TAG content (+31%), but no change in DAG and CER levels has been observed in lean animals (Benton et al., 2010). Paradoxically, robustly overexpressed PGC-1α promoted the development of IR in the skeletal muscle of the sedentary MPGC-1α TG mice, presumably as a consequence of imbalanced lipid uptake and oxidation (Summermatter et al., 2013). Excessive acyl-CoA incorporation into TCA cycle and OXPHOS was reflected in an enhanced acylcarnitine level. Moreover, the animals overexpressing PGC-1α that were fed with a high fat diet (HFD) exhibited partly different features of a lipid profile from the above mentioned, revealing no significant changes in TAG and CER content, but elevated DAG level. In addition, an increased sphingosine concentration in MPGC-1α mice resulted in a diminished glucose influx and aggravated insulin signal transduction (Summermatter et al., 2013) *via* PKB/Akt inactivation (Taha et al., 2006). Importantly, insulin sensitivity was improved when elevated level of PGC-1α was combined with exercise (Summermatter et al., 2013). On the other hand, Choi et al. observed that after PGC-1α stimulation (mRNA ≈+6-fold) HFD-induced IR occurred due to an increased membrane/cytosol DAG ratio as well as diminished Akt2 and PKCθ activity along with a reduced (−60%) insulin-stimulated glucose influx. The paralleled unequal growth in mitochondria number (+2-fold) and activity (+60%) implies the reduced activity per unit of mitochondrial mass, inevitably linking increased FA uptake with IMCL accumulation and impaired insulin tolerance (Choi et al., 2008). Some studies point out that this may be a consequence of PGC-1α/PPAR-α dependent stimulation of mammalian tribbles homolog 3 (TRB-3), which can bind to Akt1 and Akt2 and inhibit their activation (Mortensen et al., 2006). Furthermore, the unexpected exacerbation of IR in the case of muscle-specific PGC-1α upregulation (10–13-fold) has been also linked with downregulated GLUT4 mRNA level and glucose uptake. This was also supported by the observed lack of changes in GLUT-4 level in tisssues (heart muscle, white and brown adipose tissue), where PGC-1α content was not altered (Miura et al., 2003). Collectively, the alterations in lipid content and composition occur as a consequence of PGC-1α-mediated increase in gene expression of the proteins involved in both lipid oxidation and synthesis. Modestly elevated PGC-1α level accelerates the positive impact of exercise on glucose homeostasis, increases activity of enzymes involved in Krebs cycle and favors the shift from incomplete to complete β-oxidation. However, the oversized expression of the coactivator has been found to trigger mostly unfavorable metabolic changes.

## Fatty acid transporter proteins

The rate of LCFA deposition/oxidation in tissues is strictly related to the accompanying changes in their movement across both plasma and mitochondrial membranes. For many years, it was believed that the transport of FA across the plasma membrane occurs only *via* passive diffusion down their electrochemical gradient. However, currently it is well-established that FA are transported into the cells also by protein-mediated mechanism. In metabolically active tissues, such as the striated muscles and adipocytes, fatty acid translocase (FAT/CD36), fatty acid transport proteins (FATP1-6) and plasma membrane fatty acid binding protein (FABPpm) have been identified as putative FA transporters. Furthermore, protein-mediated LCFA uptake is also involved in mitochondrial lipid flow and their subsequent β-oxidation, besides the well-known carnitine-palmitoyltransferase (CPT) system (Schwenk et al., 2010). Importantly, the regulation of FA transporters expression and distribution within the cell compartments is another level of PGC-1α control over lipid homeostasis. In the current paper we summarize the role of PGC-1α in the regulation of the total, plasma membrane and mitochondrial expression of FA transporters in the insulin sensitive tissues.

### The influence of PGC-1α on the total, plasma membrane, and mitochondrial fatty acid transporters content in skeletal muscle

Skeletal muscle constitute ~40% of the human body mass and serve as a substantial tissue for FA uptake (Egan and Zierath, 2013). It has been proven that facilitated LCFA transport involves a number of transporters, such as FAT/CD36, FABPpm, FATP1, FATP4, and FATP6. However, the expression of FAT/CD36 and FABPpm, but not FATP1, corresponds with the oxidative capacity of muscle cells. Therefore, higher LCFA uptake rate is found in red oxidative muscle fibers in comparison to white glycolytic fibers (Luiken et al., 1999). For this reason, it is worth pointing out that the protein amount of FABPpm, FAT/CD36 and FATP4 highly correlates with PGC-1α expression (Nickerson et al., 2009; Benton et al., 2010), which is consistent with the fact that the protein level of PGC-1α is positively associated with the oxidative fibers ratio in metabolically heterogeneous rat hindlimb muscle (Benton et al., 2008).

Summermater et al. noticed the relationship between PGC-1α mRNA level and the FA transporters' gene expression. The authors revealed that the upregulation of PGC-1α expression in mice results in the elevation of FAT/CD36, FABPpm and FATP4 transcripts abundance, but not the mRNA amount for FATP1, FATP3, and FATP6. The subsequent increase in IMCL content was observed in MPGC-1α TG mice, although a direct analysis proved that the IMCL accumulation is caused rather by *de novo* lipogenesis than cellular FA import (Summermatter et al., 2010). Interestingly, the stimulatory effect on FAT/CD36 level was significantly greater in red fibers (195%) in comparison with white muscle fibers (35%) (Table 1) (Benton et al., 2008). The concomitant growth in cellular PGC-1α and FAT/CD36 amount was also confirmed in other studies with skeletal muscle-restricted murine PGC-1α transgenic models (Wende et al., 2007; Choi et al., 2008). However, in some studies higher transporter level (FAT/CD36) did not affect TAG muscular concentration (Benton et al., 2008), while in others an enhanced TAG content was present (Summermatter et al., 2010). On the contrary, PGC-1α overexpression in cultured human skeletal muscle cells did not increase FAT/CD36 content, although elevated FAO and expression of other genes connected with lipid metabolism were observed (Mormeneo et al., 2012; Nikolić et al., 2012). Currently, it remains unresolved if PGC-1α may directly control FABPpm expression, because there are some discrepancies in the literature as to whether the PPARγ activator rosiglitazone stimulates the increase in FABPpm content (Coort et al., 2005; Benton et al., 2008). Surprisingly, increased (≈+31%) palmitate β-oxidation may not be a consequence of lipid transport as a trend for a reduction (−6%) in the intracellular palmitate influx was observed in PGC-1α overexpressing C2C12 myotubes (Espinoza et al., 2010).

Interestingly, no significant differences in the whole muscle FAT/CD36, FABPpm and FATP4 expression has been detected between lean and obese individuals (Holloway et al., 2007), although HFD downregulates both PGC-1α and OXPHOS genes (Sparks et al., 2005). Moreover, higher TAG content in obese Zucker rats lowered PGC-1α protein expression. Conversely, mice lacking FAT/CD36 activity a parallel reduction in the number of intramuscular triacylglycerol (IMTG) depots exhibited increased PGC-1α expression. These unexpected changes suggest that the intramuscular lipid milieu may evoke alterations in the coactivator's amount independently of plasma FFA concentration (Benton et al., 2006). Additionally, the effectiveness of training-induced PGC-1α ability to stimulate the downstream genes involved in lipid utilization was impaired in FAT/CD36-knockout mice, implying an important role of FA availability in PPAR targets transcription (Manio et al., 2017). Nevertheless, the suppression of plasma FFA level *via* nicotinic acid (NA) corresponded with an increased muscle PGC-1α content without any changes after exercise. Simultaneously, the response of its target gene, FAT/CD36, did not differ between NA treated subjects and the control ones (Watt et al., 2004).

Additionally to the previous statements, Choi et al. demonstrated a paradoxical increase in IR observed in MPGC-1α TG mice (a 6-fold increase in mRNA for PGC-1α) fed with HFD, which was linked with a strong concurrent upregulation of FAT/CD36 expression (a 3-fold increase in mRNA) and FA oversupply. Moreover, the authors noticed enhanced transcription of acyl-CoA:diacylglycerol acyltransferase 1 (DGAT1) and mitochondrial glycerol-3-phosphate acyltransferase (mtGPAT), enzymes that catalyze TAG reestrification, as well as subsequent DAG and long-chain fatty acyl-CoAs accumulation (Choi et al., 2008). Furthermore, exceessive lipids provision combined with insufficient citrate synthase (CS) activity are attributed to an increased acetylcarnitine level. This in turn may deteriorate glucose metabolism *via* activation of NF-κB, which enhances insulin-stimulated phosphorylation of IRS and diminishes insulin-stimulated phosphorylatation of PKB/Akt (Zhang et al., 2010). Adverse effects of PGC-1α on insulin sensitivity have been connected with vascular endothelial growth factor β (VEGF-β) dependent stimulation of FATP3 and FATP4 expression and downstream lipid accumulation in HFD-fed MPGC-1α TG mice (Jang et al., 2016; Mehlem et al., 2016). PGC-1α also induces branched-chain amino acids (BCAA) catabolism and subsequent 3-hydroxyisobutyrate (3-HIB) secretion from muscle, resulting in excessive trans-endothelial FA transport into the tissue, deposition of lipotoxic intermediates and impaired insulin signal transmission (Jang et al., 2016). In contrast to the aforementioned findings, a limited PGC-1α-mediated FAT/CD36 upregulation (~25%) elevates insulin sensitivity (Benton et al., 2008, 2010) by stimulating FAO (Nickerson et al., 2009). It is also presumed that the mechanisms responsible for IMCL accumulation in endurance trained athletes differ from those in the individuals with T2DM. Interestingly, in the first case IMTG *de novo* synthesis prevails, whereas in the latter situation an increased FA uptake is observed (Bergman et al., 2010). Moreover, given the enhanced lipogenesis, lipid turnover seems to be insufficient in sedentary state after ectopic PGC-1α overexpression, despite enhanced oxidative capacity. This is in contrast to an active animal, where energy requirements and provision are tightly coordinated (Summermatter et al., 2010). However, the usage of PGC-1α activator, pioglitazone (thiazolidinedione), reverses disruption of insulin signaling and restores mitochondrial bioenergetics (Pagel-Langenickel et al., 2008). Thereby, a modest overexpression of PGC-1α, which restricts FAT/CD36 upregulation, seems to be a key factor for improvement of insulin sensitivity (Benton et al., 2010). On the basis of these observations, PGC-1α is regarded as one of the factors eliciting FA uptake, which rises a possibility to control their oxidation and esterification in skeletal muscle. However, its therapeutic potential may be questioned, since physiological and supraphysiological PGC-1α doses did not reverse a decreased FAO and mtDNA content in myocytes from extremely obese subjects. Therefore, skeletal muscle oxidative capacity in obesity may be partially independent of PGC-1α (Consitt et al., 2010). Currently available literature data unfortunately do not provide information about the relationship between PGC-1α expression and sarcolemmal content of FA binding and transporting proteins. To date, metabolic challenges with insulin and muscle contraction have been shown to induce the translocation of FAT/CD36 and FABPpm to the plasma membrane, thus enhancing FA delivery into muscle cells (Han et al., 2007; Bradley et al., 2012). Moreover, researchers indicate that FAT/CD36, but not FABPpm, can be constantly relocated to the plasmalemma in IR (T2DM and obesity) human skeletal muscle, whereas its total expression seems to remain unaltered (Aguer et al., 2011).

Due to the prominent impact of PGC-1α on mitochondrial expression of the key genes involved in lipid metabolism, it is also relevant to answer whether this coactivator may exhibit a pronounced effect on protein mediated FA delivery to this organellum. So far, most of the studies have demonstrated that PGC-1α can increase the expression of enzymes like carnitine palmitoyltransferase 1b (CPT1b), carnitine-acylcarnitine translocase and malonyl-CoA decarboxylase that are implicated in the long-chain fatty acyl-CoA delivery for β-oxidation (Espinoza et al., 2010; Gacias et al., 2012). In addition, modest overexpression of PGC-1α was associated with elevated FAT/CD36 content in the mitochondrial skeletal muscle fraction from transfected mice. However, such an effect was observed solely within the SS mitochondria (+15–17%), but not in the IMF (Benton et al., 2008). On the contrary, pharmacological PGC-1α downregulation (i.e., clonbuterol administration) and the concomitant reciprocal increase in RIP140 expression evoked a reduction in FAT/CD36 total and, specifically, the IMF mitochondrial protein content in both red (−36 and −36%, respectively) and white (−35 and −58%, respectively) skeletal muscle. Accordingly, the alterations in mitochondrial content and structure together with a reduced expression of the enzymes responsible for FAO were also attributed to PGC-1α decrease (Hoshino et al., 2012). In regard to PGC-1α ability for enhancing cellular oxidative capacity, palmitate utilization was stimulated selectively in the SS mitochondria of both red (+116%) and white (+40%) muscles (Benton et al., 2008). These observations may reflect the fact that palmitate oxidation in both the mitochondrial subpopulations was higher in red muscle fibers (Koves et al., 2005a), although the oxidative capacity of the IMF type was elevated in comparison with the SS mitochondria (Ferreira et al., 2010). Moreover, the rate of palmitate oxidation in the IMF mitochondria did not differ between lean and obese Zucker rats, but the oxidation in the SS subpopulation was higher in the case of obese animals (Holloway et al., 2009). Novel findings revealed that the absence of fat overload in muscles of T2DM rats ensures normal function of the SS and IMF mitochondria (Lai et al., 2017). A comprehensive proteomic analysis confirmed an upregulation of FAT/CD36 and FABP3 (heart-type FABP, H-FABP) protein abundance as well as intensified the rate of β-oxidation in the mitochondria isolated from skeletal muscle of transgenic PGC-1α mice on a HFD. Moreover, exercise and caloric restriction declined the acylcarnitines level in the muscle of these animals, although the opposite response was observed in the case of 18:1 and 18:2 DAG species (Wong et al., 2015). Importantly, the transgenic mitochondria rich in PGC-1α can tolerate greater level of FA supply as well as exhibit an enhanced level of maximal uncoupled respiration (Hoeks et al., 2012). The presented findings support the hypothesis that PGC-1α induces mitochondrial adjustments aimed to cope with a disproportionate lipid delivery.

In summary, the proposed mechanisms of PGC-1α involvement in the rate of lipid movement into skeletal muscle include an upregulation of FAT/CD36, FABPpm and FATP1 gene expression as well as protein content in mitochondrial fraction. The latter effect has been confirmed solely in the case of murine FAT/CD36 and FABP3. However, it should be emphasized that the coactivator's insulin-sensitizing role is connected specifically with limited, i.e., within physiological range, PGC-1α overexpression. The used model also plays a vital role in the evaluation of PGC-1α effects, since the lack of stimulatory effect on FA transporters content was observed in human cultured myocytes. Moreover, insufficient information gives rise to the doubts, whether PGC-1α possesses the ability to relocate FA transporters to the sarcolemma or what are the direct molecular mechanisms responsible for the aforementioned changes.

### The influence of PGC-1α on the total, plasma membrane, and mitochondrial fatty acid transporters content in the heart

It is well-documented that under physiological conditions 70–80% of myocardial energy expenditure are covered by β-oxidation of FA, however the rate of LCFA influx depends mainly on their plasma amount (Chabowski et al., 2008). Additionally, the FA transport into isolated cardiomyocytes can occur against the concentration gradient, which was measured by acrylodan labeled intestinal fatty acid binding protein (ADIFAB) (Carley and Kleinfeld, 2011). Importantly, the high PGC-1α content in the heart is connected with some specific features in comparison with other insulin sensitive tissues. For instance, a significant increase in PGC-1 expression is observed at birth in conjunction with an increase in cardiac oxidative capacity and a shift from glucose toward FA as a fundamental energy source during postnatal development (Buroker et al., 2008). Moreover, in the heart muscle the coactivator's mRNA level rises after fasting with no changes observed in BAT (Russell et al., 2004).

A causal relationship between PGC-1α/PPARα activation and an enhanced expression of multiple mitochondrial ATP-generating pathways was supported by animal model study (Duncan et al., 2007). PGC-1α increases the levels of some critical enzymes involved in β-oxidation, including the components of the OXPHOS complex and TCA cycle (Table 1) (Rowe et al., 2010). The expression of FA transporters is also controlled by myocardial PPARs, since the presence of peroxisome proliferator-responsive elements (PPREs) has been identified in the FAT/CD36, FABPpm, and FATP promoters sequence (Frohnert et al., 1999; Teboul et al., 2001). In accordance with this notion, the mRNA levels for FATP1 and FAT/CD36 were significantly blunted in PPARα-null mice as compared to wild type animals. Simultaneously, radioactively labeled palmitic acid uptake and oxidation were decreased due to lower expression of FA transporters (Watanabe et al., 2000). On the other hand, cardiac-restricted overexpression of PPARα in mice did not alter the transcription of FATP1 and FAT/CD36 in basal conditions, although PPARα agonist treatment (Wy-14,643) induced cardiac expression of these FA transporters (Finck et al., 2002). Moreover, the exposure to high concentration of FA coordinately enhanced mRNA content for FAT/CD36 and FABPpm as well as increased cellular oxidative capacity (van der Lee et al., 2000). PGC-1α interaction with ERRα also contributes to the regulation of cardiac lipid metabolism and upregulation of FAT/CD36, FABP3, and lipoprotein lipase (LPL) genes (Huss and Kelly, 2004; Huss et al., 2004). Huss et al. additionally reported a 92% increase in palmitate oxidation in PGC-1a overexpressing cells. Moreover, the compensatory acceleration in PGC-1α content in ERRα null mice, prevented FAT/CD36 and CPT I reduction (Huss et al., 2004).

Some authors speculate that the upregulation of the nuclear receptor transcription factor PPARα and its coactivators PGC-1α/β is one of the preliminary requirements for metabolic switch from glucose to FA consumption in IR and diabetic hearts (Schilling, 2015; Lee et al., 2017). This agrees with an increased level of PPARα as well as the total (+38%) and sarcolemmal (+26%) FAT/CD36 content in the diabetic animal hearts (Mansor et al., 2017). On the contrary, lowered PGC-1α expression was observed in diabetic human blood and myocardium (Fabregat-Andres et al., 2016) as well as in the animal models of T2DM (ob/ob mice) and diabetic dyslipidemia (db/db mice) (Buchanan et al., 2005). PPARα determines the expression of enzymes controlling both the catabolic and anabolic pathways of TAG metabolism (Banke et al., 2010). Cardiac-specific PPARα overexpression induced the occurrence of the hallmarks of diabetes, such as diminished glucose oxidation and increased cardiomyocyte TAG storage (Yang et al., 2007). Simultaneously, an elevated turnover of palmitoyl-CoA between cytosolic and TAG pool prior to β-oxidation and augmented recruitment of TAG-derived LCFA for utilization were observed in MHC-PPARα mice (Banke et al., 2010). Interestingly, PPARα and FAT/CD36 deficient mice [α-myosin heavy chain (MHC)-PPARα/CD36^−/−^] were not only protected from lipid imbalance in the myocardium, but also demonstrated an enhanced glucose uptake and FATP1 gene expression (Yang et al., 2007). Nevertheless, direct inhibition of plasma membrane FAT/CD36 by sulfo-N-succinimidyl oleate was sufficient to restore normal substrate metabolism and prevent lipotoxicity (Mansor et al., 2017). PPARγ overexpression in transgenic mice contributed to cardiac dysfunction encompassing an increase in FAT/CD36 level, FA uptake and intracellular lipid accumulation with rosiglitazone treatment even aggravating the lipotoxic effects. On the contrary, the action of rosiglitazone in the heart of wild type mice has been connected with a decreased expression of PPARγ target genes and a redistribution of PPAR ligands (i.e., plasma lipids) mainly to adipose tissue (Son et al., 2007). The consequences of PPARβ/δ knockout included a decreased PGC-1α protein content together with diminished FABP3, FATP1 and CPTIb genes expression in the adult mouse heart (Wang et al., 2010). The cardiomyocytes of MHC-PPARβ/δ mice were characterized by increased glucose uptake and utilization, induced FAO genes expression, lack of a stimulating effect on FA uptake and lipogenesis (with no signs of abnormal lipid accumulation) and, as a result, lipotoxic cardiomyopathy did not develop (Burkart et al., 2007).

Most of the studies corroborate the presence of FA transporters in both plasma membrane and the low-density microsome compartment of cardiomyocytes (Chabowski et al., 2005). Chronic translocation of FAT/CD36 and FABPpm to the plasma membrane, as a mechanism underlying TAG accumulation, was shown in the hearts from obese Zucker rats (Luiken et al., 2001; Coort et al., 2004) as well as in moderate and severe streptozotocin-induced diabetes of Sprague-Dawley rats (Luiken et al., 2002). Experiments in our laboratory revealed that chronic activation of PPARα induces the redistribution of FAT/CD36 from low density microsomes to the plasma membrane along with the increased palmitate incorporation into DAG fraction. PPARβ/δ stimulation is responsible for the relocation of both FAT/CD36 and FABPpm to the plasmalemma. Moreover, an enhanced level of myocardial TAG synthesis and palmitate oxidation was observed after PPARβ/δ agonist treatment. On the contrary, the usage of PPARγ activator did not exert any influence on the intracellular localization of these transporters as well as intracellular lipid pools content, although a decreased level of palmitate oxidation was noticed (Kalinowska et al., 2009).

Mitochondria constitute 20–30% of a cardiomyocyte volume, therefore they are substantial for the maintenance of an optimal FAO rate (Dillon et al., 2012). However, little is known regarding the possibility of FA transporters relocation into an out of cardiac mitochondrial membranes. Currently, solely the presence of FAT/CD36 has been confirmed. Interestingly, the reported mitochondrial expression of FAT/CD36 in the heart was ~10-fold greater than the one observed in red and white skeletal muscle (Campbell et al., 2004; King et al., 2007). Moreover, in the hearts of mice FAT/CD36 depletion did not affect the rate of palmitate, palmitoyl-CoA and palmitoylcarnitine mitochondrial β-oxidation, indicating considerably unessential role of this carrier in the mitochondrial lipid utilization in that tissue (King et al., 2007).

In conclusion, PGC-1α may play a key role in orchestrating FA supply to the heart *via* PPARs coactivation. However, potentially beneficial therapeutic window for PGC-1α in the cardiomyocytes is relatively narrow, since its overexpression leads to uncontrolled mitochondrial proliferation, abnormal sarcomeric structure and dilated cardiomyopathy (Lehman et al., 2000; Lehman and Kelly, 2002). Additionally, PPARα stimulation is thought to be a main culprit responsible for the excessive TAG, CER and DAG accumulation through incorporation of FAT/CD36 to the membrane structure in IR cardiomyocytes.

### The influence of PGC-1α on the total, plasma membrane, and mitochondrial fatty acid transporters content in the adipose tissue

Adipose tissue serves as a primary tissue involved in lipolysis and lipogenesis, thereby contributing to the maintenance of FA uptake and release from/to the blood. In contrast to striated muscle, the driving gradient for FA diffusion between circulation and adipocytes may not occur, what suggests differences in the regulation of protein-mediated LCFA transport between the both tissues. Indeed, the facilitated FA transfer across plasma membrane constitutes more than 90% of their total flux in fat tissue (Stump et al., 2001). During their entry to the cell FA are accompanied by adipocyte cytoplasmic FABP (A-FABPc) that traffics them to a specific compartment and thereby decides about their further destination (Furuhashi and Hotamisligil, 2008). Pivotal transporters governing LCFA uptake into adipocytes are FAT/CD36, FABPpm, FATP1, and FATP4. Moreover, continuous recycling of these proteins from intracellular pool to the plasma membrane guarantees an appropriate plasma lipid concentration. In view of a significant role of FA in energy homeostasis, disturbances in the incorporation of LCFA into adipocytes may lead to IR in obesity and T2DM.

PGC-1α controls multiple morphological and molecular features required for the differentiation and proper function of brown adipocytes. It has been demonstrated that PGC-1α content is greater in subcutaneous than in omental adipose tissue regardless of obesity and fat distribution. The mRNA level for the coactivator inversely correlates with augmented body fat mass, visceral fat accumulation, as well as impaired glucose and insulin tolerance (Ruschke et al., 2010). Additionally, PGC-1α expression is significantly decreased in the subcutaneous adipose tissue of non-obese, non-diabetic IR individuals (Hammarstedt et al., 2003) and morbidly obese subjects (Semple et al., 2004).

Mice with adipose-restricted PGC-1α deficiency exhibited multiple alterations, including decreased lipid clearance arising as a result of FA transporter (FABP3), OXPHOS, TCA cycle and FAO genes downregulation. However, apart from FABP3 these observations affected solely WAT, while in BAT no significant changes occurred. Similar reduction (−20–40%) in mitochondrial genes expression was demonstrated in the cultured adipocytes derived from the inguinal stromal-vascular fraction (Kleiner et al., 2012). HFD fed mice lacking PGC-1α are characterized by increased FFA and TAG plasma levels along with decreased transcription of the genes involved in FAO as well as lipogenesis. Moreover, their FA uptake and breakdown were impaired as a consequence of reduced gene expression of FABP3 by 50%, FAT/CD36 by 30–40%, LPL and lipoprotein(a) in BAT (Kleiner et al., 2012). In WAT only a downward trend for FAT/CD36 and LPL transcripts content has been revealed. In both types of adipose tissue no changes in FATP1 mRNA level were noticed in response to PGC-1α ablation (Kleiner et al., 2012). As observed, dysregulation of genes involved in FA uptake, oxidation and synthesis strongly affected whole-body insulin sensitivity, mainly by increased hepatic glucose output (Kleiner et al., 2012). Interestingly, the knockout of adipocyte FABP (FABP4) reduced the potency of HFD for the increase in PGC-1α in BAT, while FAT/CD36 and FATP1 gene expression was upregulated to a similar extent as in a wild type mice (Shu et al., 2017). The role of PGC-1α in WAT has been questioned by Pardo et al. since the authors had not found significant differences in the expression of the genes involved in FA transport (FAT/CD36) and metabolism in adipocyte-restricted PGC-1α knockout mice (Pardo et al., 2011). Additionally, rosiglitazone treatment of these animals improved peripheral insulin sensitivity indicating that PGC-1α activity in WAT is not crucial for the whole body glucose and lipid balance. Furthermore, rosiglitazone effectively stimulated the expression of OXPHOS, TCA, and FAO genes, implying non-essential PGC-1α function in the mitochondrial biogenesis in WAT. Therefore, Pardo et al. performed studies on 3T3-L1 adipocytes and demonstrated that PGC-1β, instead of PGC-1α, acts as a primary modulator of the mitochondrial function in WAT, whereas PGC-1α is more important in the induction of brown adipocyte markers in white fat cells (Pardo et al., 2011). Surprisingly, Lin et al. showed that mice with whole-body PGC-1α ablation are resistant to HFD-induced obesity as well as exhibit increased insulin sensitivity and ameliorated glycemic control as indicated by glucose tolerance test (Lin et al., 2004). These findings are similar to the observations obtained by Leone et al. in PGC-1α^−/−^ mice, which were less susceptible to HFD-induced insulin resistance, despite an increased body fat mass (Leone et al., 2005). Additionally, accumulating evidence suggest that the hypoglycemic effect exerted by the activation of AMPK/PGC-1α axis (metformin) may be of secondary importance, since AMPK deletion did not abolish metformin's action (Viollet et al., 2012). Nevertheless, high basal PGC-1α and FABP4 expression in adipose tissue are the predictors of good treatment response to pioglitazone, independently of the changes in PGC-1α mRNA level (Hammarstedt et al., 2008).

To summarize, the collected data corroborate the fact that FA transporters translocate to the cell membranes in the adipose tissue of obese Zucker rats. However, PGC-1α contribution to the regulation of their expression and cellular localization remains undermined or described in limited extent. Nonetheless, the reduction of protein transporters content (FAT/CD36, FABP3) in BAT of PGC-1α knockout mice indicates the connection between intracellular FA transport and PGC-1α activity.

## Summary

Inefficient utilization and excessive lipids accumulation in tissues consequently result in a disruption of the insulin signaling pathway. The complexity of interactions between FA delivery to the insulin-sensitive tissues and their subsequent downstream metabolism makes it difficult to find one obvious target that may enable to control the rate of these processes. Therefore, the role of PGC-1α in modulating the activity and cellular localization of FA transport proteins (FAT/CD36, FABPpm, FATP1-6) has attracted considerable attention. This coactivator is widely known to increase the expression of critical genes involved in OXPHOS and TCA cycle, but also in lipid synthesis (i.e., FA, DGAT1, CS). So far, there is a limited number of studies linking PGC-1α protein expression (down- and upregulation models) with the total, membrane and mitochondrial content of FA transport proteins in insulin sensitive tissues. As previously shown, PGC-1α upregulates both protein expression and mitochondrial content of LCFA transporters in skeletal muscle, thereby modulating the rate of lipid oxidation. Thus, PGC-1α-stimulated FA targeting for mitochondrial β-oxidation may diminish the rate of FA storage and the synthesis of lipotoxic derivatives. On the other hand, despite much progress in describing cardiac- and adipocyte-specific coactivator's effects, its influence on FA protein transporters has yet to be elucidated. In conclusion, regulation of the expression of FA transporters may indicate PGC-1α as a prospective target for the prevention and/or treatment of IR and, as a consequence, of T2DM.

## Author contributions

ES participated in the design of the work, drafted the manuscript and approved the final version submitted. AM provided substantial contributions to the conception and design of the work, helped to draft the manuscript and approved the final version submitted. AC participated in the design of the study, revised it critically for important intellectual content and approved the final version submitted. All authors agree to be accountable for all aspects of the work.

### Conflict of interest statement

The authors declare that the research was conducted in the absence of any commercial or financial relationships that could be construed as a potential conflict of interest. The reviewer DV and handling Editor declared their shared affiliation.
